# Noninvasive prenatal testing detected acute myeloid leukemia in paucisymptomatic pregnant patient

**DOI:** 10.1002/ccr3.3027

**Published:** 2020-06-20

**Authors:** Laura Yissel Rengifo, Lucienne Michaux, Johan Maertens, Thomas Tousseyn, Liesbeth Lenaerts, Joris Robert Vermeesch, Peter Vandenberghe, Barbara Dewaele

**Affiliations:** ^1^ Center for Human Genetics University Hospitals Leuven Leuven Belgium; ^2^ Department of Microbiology and Immunology KU Leuven Leuven Belgium; ^3^ Department of Hematology University Hospitals Leuven Leuven Belgium; ^4^ Department of Pathology University Hospitals Leuven Leuven Belgium; ^5^ Department of Oncology KU Leuven Leuven Belgium

**Keywords:** acute myeloid leukemia, bone marrow dry tap, hematology, incidental finding, malignancy, noninvasive prenatal testing

## Abstract

To the authors' best knowledge, this is the first report of acute myeloid leukemia (AML) detected by noninvasive prenatal testing. This was an aggressive case that otherwise would have been difficult to characterize due to disadvantages of "gold‐standard" techniques.

## Graphical abstract

To the authors' best knowledge, this is the first report of AML detected by noninvasive prenatal testing. This was an aggressive case that otherwise would have been difficult to characterize due to disadvantages of "gold‐standard" techniques.

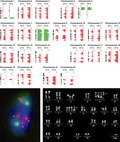

## 1 INTRODUCTION

Noninvasive prenatal testing (NIPT) is generally applied as screening method during pregnancy. Using next‐generation sequencing (NGS) of circulating cell‐free DNA (ccfDNA) from peripheral blood (PB) plasma, NIPT can detect common fetal‐derived chromosomal aneuploidies. Additionally, maternal copy number aberrations (CNAs) can be identified.^1^ The latter may represent either an unrecognized constitutional modification, or a presymptomatic acquired maternal malignancy.

Diagnosis of an acquired malignancy during pregnancy can be difficult since some of the symptoms related to cancer, like fatigue or nausea, are very similar to those of pregnancy.^2^ For several types of cancer, it was already shown that NIPT is able to detect cancer‐related genetic aberrations in the presymptomatic stage.^2^, ^3^ This holds the promise of accelerating the diagnosis of maternal malignancy and improving long‐term maternal and neonatal outcomes.^1^, ^2^

The current study describes the incidental finding of acute myeloid leukemia (AML) in a pregnant woman following aberrant NIPT result.

## 2 PATIENT CHARACTERISTICS AND DIAGNOSIS

**Figure 1:**
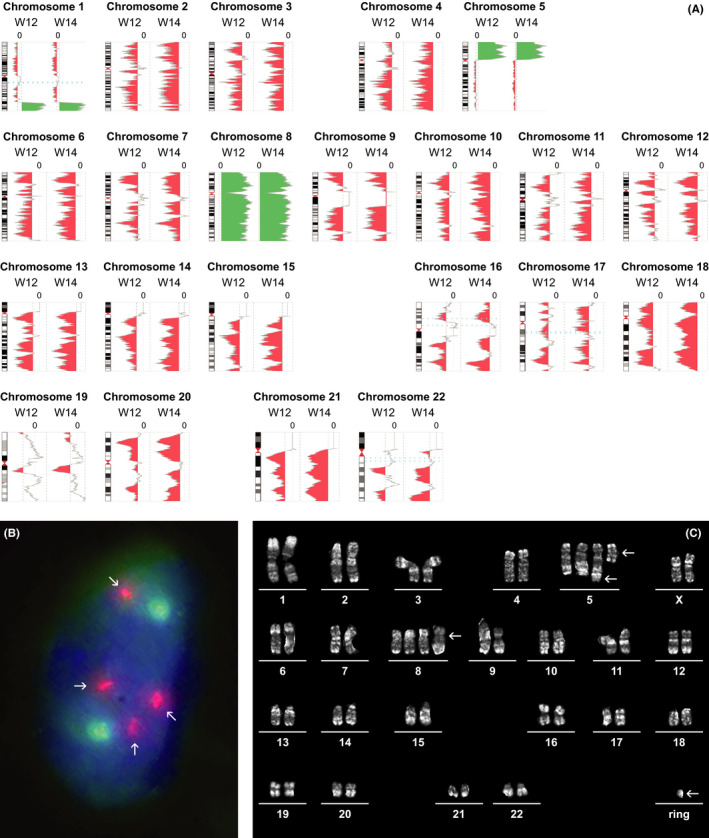
NIPT^a^ profile during pregnancy with partial imbalances in chromosomes 1 and 5 and gain of chromosome 8. A, The chromosome ideogram is at the left with the profile of each NIPT test (week 12 and week 14) at the right; green represents a likely duplicated or amplified region and red represents a likely deleted region; dotted lines on either side of the axis, plus or minus 1.5×. B, Interphase FISH^b^ on peripheral blood, using a centromeric probe for chromosome 8 (CEP8 (SO) [8p11.1‐q11.1, Vysis]) and as reference a centromeric probe for chromosome 9 (XCE 9 (Green) [9p11.1‐q11.1, Metasystems]), proves the presence of tri‐ or tetrasomy 8 (arrows) at the time of AML diagnosis. C, Representative R‐banded metaphase showing a complex karyotype (400 bphs^c^). Unbalanced t(1;5), additional derivative chromosome 5 from the t(1;5), isochromosome 5p; tetrasomy 8 and presence of a ring chromosome (arrows). Some of these aberrations had been detected by NIPT^b^ at week 12 of the pregnancy. ^a^NonInvasive Prenatal Test. ^b^Fluorescent In Situ Hybridization. ^c^Bands per Haploid Set

The 32‐year‐old woman appeared healthy prior to her second pregnancy. She had given birth to a healthy girl by caesarean section 2 years earlier. Her medical history was otherwise unremarkable. During her second pregnancy, routine NIPT screening at week 12 showed maternal somatic aberrations consisting of +1q, +5p, and gain of chromosome 8 (Figure 1A). At week 14, she presented to consultation with complaints of severe back and abdominal pain, and discomfort in both legs. At that time, routine laboratory results were unremarkable, showing normal PB values.

Because of the abnormal NIPT profile, bone marrow (BM) examination was performed. Only a minimal quantity of BM could be obtained (dry tap), resulting in the lack of material for cytogenetics and molecular diagnostics. PB samples were analyzed instead. Immunophenotyping on PB showed a small population of myeloblasts positive for CD34, CD117, and CD13 and weak to negative for CD33. NGS showed *NPM1* (type Leu 21) as well as *PTPN11* variants (Appendix S1). Cytogenetic analysis on PB was normal at that time.

Whole‐body diffusion magnetic resonance showed lesions in liver, spleen, and BM, suggesting infiltration by a hematological neoplasm. BM trephine disclosed hypercellularity with diffuse blastic infiltration and mild fibrosis (grade 1, focal grade 2), compatible with an acute monocytic leukemia.

Elective termination of the pregnancy followed at 14 weeks.

## 3 PATIENT TREATMENT AND EVOLUTION

Induction therapy with Idarubicin/Cytarabine (12 mg/m^2^ d1‐3 and 200 mg/m^2^ d1‐7, respectively) started immediately after termination of the pregnancy. Remission was achieved. Six weeks after diagnosis, BM showed minimal amount of blasts (3.3% by morphology; less than 0.7% by immunophenotype), and *NPM1* mutated transcripts were molecularly undetectable (Table S1).

Consolidation with high dose Cytarabine (1g/m^2^ 2×/d for 6 days) was given. Morphological remission was achieved, whereas immunophenotype showed a small amount of myeloblasts (<0.1% of ANC). At this point, monitoring of *NPM1* transcripts showed a 1 log increase compared with follow‐up sample 1 (postinduction).

The patient proceeded to Busulfan (16 mg/kg) and Cyclophosphamide, followed by autologous peripheral stem cell transplantation. Partial remission was achieved. Cytogenetic examination of BM was normal, and molecular analysis showed a decrease (>3 log) of the number of positive cells for *NPM1* mutation in comparison with the previous analyzed BM sample (Table S1).

Relapse occurred seven months after diagnosis and was refractory to reinduction therapy (high dose Cytarabine), followed by Venetoclax and Decitabine. The patient died 10.5 months after diagnosis.

## 4 NIPT AND CYTOGENETICS

The aberrant NIPT profile at week 12 of the pregnancy, prompted a second NIPT at 14 weeks. The identical profiles reinforced the presence of +1q, +5p, and gain of chromosome 8 in ccfDNA.

Because of dry tap, interphase FISH was performed on PB, confirming tri‐tetrasomy 8 in approximately 20% of the cells: nuc.ish(D8Z2 × 3 ~ 4,D9Z1 × 2 ~ 3)[19/100] (Figure 1B). Routine FISH for *KMT2A* [11q23] and *MECOM* [3q26] showed no aberrations (nuc.ish(MECOM × 2)[99/100],(KMT2A × 2)[99/100]).

At diagnosis, karyotype on PB was normal: 46,XX[20], as well as four follow‐up BM karyotypes. At relapse, 7 months after diagnosis, PB cytogenetics revealed a complex karyotype, including tetrasomy 8, a translocation between chromosomes 1 and 5, an isochromosome of the short arm of chromosome 5 and a ring chromosome of unknown origin: 51,XX,der(5)t(1;5)(q41;q3?4),+der(5),+i(5)(p10),+8,+8,+r[2]/46,XX[9] (Figure 1C). This karyotype matched NIPT results at 12/14 weeks of pregnancy.

## 5 DISCUSSION AND CONCLUSIONS

While it is well established that NIPT can unveil maternal cancer that otherwise might have remained undetected until later stages of the malignancy,^1^ to the best of our knowledge, this is the first report of AML incidentally detected by NIPT in a pregnant woman.

Acute myeloid leukemia is a hematological malignancy characterized by the presence of clonal myeloid blasts in PB, BM, and/or other tissues ^4^; it is usually diagnosed by the presence of more than 20% blasts in PB or BM^5^ but presence of some specific genetic aberrations can be sufficient to establish the diagnosis.^4^ The disease presents with one of more of the following: cytopenia, fatigue, gum hemorrhages, and/or infections with fever.^6^ Usually, the incidence of AML increases with age, being more common in patients >65 years ^4^, ^6^; it is very rare and life‐threatening during pregnancy,^7^ occurring once in about 75 000‐100 000 pregnancies, with an overall survival of approximately 30%.^8^ Management of the disease is challenging, resulting in some late interventions.^7^

In AML, knowledge of the genomic profile is important as recurrent genomic abnormalities have prognostic significance. AML with mutated *NPM1* is often observed in association with normal karyotype and typically has a better outcome.^8^ However, 5%‐15% of AML with mutated *NPM1* show chromosomal aberrations, including recurrent trisomy 8 and del(9q). Trisomy 8, found in 10%‐15% of cases, carries intermediate prognosis that could be “worsened” when associated with other abnormalities.^9^ In our patient, FISH on PB validated gain of chromosome 8. At relapse, all aberrations detected with NIPT at diagnosis were confirmed and further specified by the karyotype, for example, the previously observed +1q and +5p resulted from an unbalanced translocation between chromosomes 1 and 5. In addition, extra 5q and a ring chromosome were identified. This clonal evolution resulted in a complex hyperdiploid clone.

Individuals with AML are usually treated with Anthracycline‐Cytarabine‐based regimens that can induce and/or select additional DNA mutations (like induced transversion‐type mutations), producing possible new clones. Frequently, these complex modifications confer an advantage onto later clones, enabling treatment resistance associated with relapse.^10^ In our patient, due to dry tap at diagnosis, the aberrant karyotype could only be detected at relapse, after intensive treatment. Delayed detection of these aberrations likely demonstrates clonal evolution characteristic of AML.

In summary, our case illustrates that NIPT analysis on PB offers a powerful technique for early detection of asymptomatic malignancies during pregnancy. Additionally, our findings highlight the potential of low coverage sequencing ccfDNA for routine diagnostic workup of hematological malignancies, including AML, especially in the event of dry tap, or failed karyotype.

## CONFLICT OF INTEREST

None declared.

## AUTHOR CONTRIBUTIONS

LYR: wrote the manuscript. LM: supervised the cytogenetic and molecular procedures, the analysis of the results and revised the manuscript. JM: followed the patient and revised the manuscript. TT: followed the patient and revised the manuscript. LL: revised the manuscript. JRV: revised the manuscript. PV: followed the patient and revised the manuscript. BD: supervised the cytogenetic and molecular procedures, the analysis of the results and revised the manuscript.

## Supporting information

Table S1

Appendix S1
