# Supplementary material for: Noninvasive prenatal testing detected acute myeloid leukemia in paucisymptomatic pregnant patient
Source: Clin Case Rep. 2020 Jun 20;8(10):1924–7. doi: 10.1002/ccr3.3027 (PMC7562837; doi:10.1002/ccr3.3027)
Supplement: Supplementary file 1 — Table S1 [file CCR3-8-1924-s001.docx]

**Supplementary Table 1.** Evolution ratio of mutant *NPM1/ABL1*. *Relative quantification

| **Sample** | **Weeks after diagnosis** | **Sample Type** | ***NPM1/ABL1* Ratio*** |
| --- | --- | --- | --- |
| Diagnosis | 0 | Blood | 31,60 |
| Follow-up sample 1 | 6 | Bone Marrow | 0.00 |
| Follow-up sample 2 | 17 | Bone Marrow | 1,10 |
| Follow-up sample 3 | 24 | Bone Marrow | 0,00053 |
| Follow-up sample 4 | 30 | Blood | 13,17 |
