# Supplementary material for: Noninvasive prenatal testing detected acute myeloid leukemia in paucisymptomatic pregnant patient
Source: Clin Case Rep. 2020 Jun 20;8(10):1924–7. doi: 10.1002/ccr3.3027 (PMC7562837; doi:10.1002/ccr3.3027)
Supplement: Supplementary file 2 — Appendix S1 [file CCR3-8-1924-s002.docx]

**Supplementary methods**

**NIPT**

A peripheral blood sample was obtained using Cell-Free DNA BCT tubes (Streck, Omaha, NE, USA). ccfDNA was extracted starting from 4 mL to 5 mL of plasma. The resulting ccfDNA was used for the preparation of sequencing libraries with the TruSeq Chip Sample preparation kit (Illumina, San Diego, CA, USA). Multiplex sequencing was done across both lanes of an Illumina HiSeq2500 (fast mode producing 50 bp single end reads in pools of 23–24 samples) (1). Reads were processed, corrected and analyzed with Burrows-Wheeler aligner, Picard instrument, and Genome Analysis Toolkit. Per chromosome, a Z-score was calculated based on the genomic representation of a reference set of normal samples, to establish gains or losses.

**FISH**

At diagnosis, fluorescent *in situ* hybridization was performed on a cytospin from fresh peripheral blood according to the protocols described in Put *et al.*(2) In short, the slides were fixed with Carnoy mix (3 parts methanol per 1 part glacial acetic acid) and hybridized with the probes CEP8 (SO) [8p11.1-q11.1, Vysis-Abbott Molecular Inc., Des Plaines, IL], XCE 9 (Green) [9p11.1-q11.1, Metasystems Inc., Boston, MA.], LSI MLL (DC BA) [11q23, Vysis] and XL MECOM (DC BA) [3q26, Metasystems]. Signals were evaluated in 20 to 200 nuclei, images were taken and stored using Isis fluorescence imaging system (MetaSystems Inc., Boston, MA.).

**Karyotype**

Bone marrow and peripheral blood samples were cultivated, incubated, spread on glass slides and stained following standard protocols for conventional cytogenetics (3). In brief, a Coulter counter (Beckman Coulter, Analis, Namur, Belgium) was used to determine the cellularity of the sample. At diagnosis, no bone marrow was available for karyotyping due to dry tap. Peripheral blood was cultured instead stimulated using a cocktail of cytokines. Two cultures, starting from a concentration of at least 1.5 x 106 cells/mL, were incubated for 24 and 48 hours respectively. At follow-up, myeloid cells from bone marrow were cultured but at relapse, again only peripheral blood was available for karyotyping. Metaphase spreads on microscopy slides were stained with R-banding; photography and analysis used the Ikaros karyotyping system software (MetaSystems Inc., Boston, MA.).

**Amplicon-based targeted resequencing (Next-Generation Sequencing)**

For amplicon-based targeted resequencing, the Illumina platform protocol was used (Illumina, Inc., San Diego, CA, USA). Briefly, a library was prepared from 50 ng of fresh DNA for targeted re-sequencing using the TruSight Myeloid Sequencing kit (Illumina, Inc., San Diego, CA, USA; detection limit: 5% mutant alleles; 10% tumor cells) and sequencing libraries were subjected to a MiSeq sequencer (Illumina Inc., San Diego, CA, USA) The following genes were examined for abnormalities in hotspot regions: *ABL1, ASXL1, ATRX, BRAF, CALR, CBL, CBLB, CBLC, CSF3R, FBXW7, FLT3, GATA1, GATA2, GNAS, IDH1, IDH2, JAK2, KIT, SCRATCH, MPL, MYD88, NOTCH1, NPM1, NRAS, PDGFRA, PTEN , PTPN11, SETBP1, SF3B1, SMC1A, SMC3, SRSF2, TET2, TP53, U2AF1* and *WT1*. In addition, the coding region of genes *BCOR, BCORL1, CDKN2A, CUX1, DNMT3A, ETV6, EZH2, IKZF1, KDM6A, PHF6, RAD21, RUNX1, STAG2* and *ZRSR2* were analyzed. The total panel contains 568 amplicons. NGS data analysis was performed using our in-house developed sequencing pipeline for routine diagnostics. The NGS data passed local quality control parameters. A coverage of 500x per amplicon was anticipated. For *CEBPA*, *HRAS* and *JAK3*, insufficient reads are systematically generated; no results are reported for these genes. Also, for other regions with a coverage below 500x, no results are reported. The results are given in percentages referring to the ratio of the number of mutant calls versus the total number of calls for a given position. Nomenclature of genetic variants was designated according to the Human Genome Variation Society recommendations. Classification of variants was performed according to current standards and guidelines (4,5).

**Real Time-PCR**

The following molecular assessments were done by routine quantitative RT-PCR*: DEK-NUP214, CBFB-MYH11*, and *BCR-ABL1* fusion transcripts, *FLT3-ITD, KMT2A-PTD, NPM1, CEBPA* mutations, *MECOM* overexpression (6–9).

**Supplementary References**

1. Bayindir B, Dehaspe L, Brison N, Brady P, Ardui S, Kammoun M, et al. Noninvasive prenatal testing using a novel analysis pipeline to screen for all autosomal fetal aneuploidies improves pregnancy management. Eur J Hum Genet [Internet]. 2015 Oct 22;23(10):1286–93. Available from: http://dx.doi.org/10.1038/ejhg.2014.282

2. Put N, Lemmens H, Wlodarska I, Konings P, Moreau Y, Hagemeijer A, et al. Interphase fluorescence in situ hybridization on selected plasma cells is superior in the detection of cytogenetic aberrations in plasma cell dyscrasia. Genes Chromosom Cancer. 2010 Nov;49(11):991–7.

3. Put N, Konings P, Rack K, Jamar M, Roy N Van, Libouton J-M, et al. Improved detection of chromosomal abnormalities in chronic lymphocytic leukemia by conventional cytogenetics using CpG oligonucleotide and interleukin-2 stimulation: A Belgian multicentric study. Vol. 48, Genes, Chromosomes and Cancer. 2009. p. 843–53.

4. Li MM, Datto M, Duncavage EJ, Kulkarni S, Lindeman NI, Roy S, et al. Standards and Guidelines for the Interpretation and Reporting of Sequence Variants in Cancer. J Mol Diagnostics [Internet]. 2017 Jan;19(1):4–23. Available from: https://linkinghub.elsevier.com/retrieve/pii/S1525157816302239

5. Richards S, Aziz N, Bale S, Bick D, Das S, Gastier-Foster J, et al. Standards and guidelines for the interpretation of sequence variants: a joint consensus recommendation of the American College of Medical Genetics and Genomics and the Association for Molecular Pathology. Genet Med. 2015 May;17(5):405–24.

6. Gabert J, Beillard E, van der Velden VHJ, Bi W, Grimwade D, Pallisgaard N, et al. Standardization and quality control studies of “real-time” quantitative reverse transcriptase polymerase chain reaction of fusion gene transcripts for residual disease detection in leukemia - a Europe Against Cancer program. Leukemia. 2003 Dec;17(12):2318–57.

7. van Dongen JJM, Macintyre EA, Gabert JA, Delabesse E, Rossi V, Saglio G, et al. Standardized RT-PCR analysis of fusion gene transcripts from chromosome aberrations in acute leukemia for detection of minimal residual disease. Leukemia [Internet]. 1999;13(12):1901–28. Available from: https://doi.org/10.1038/sj.leu.2401592

8. Benthaus T, Schneider F, Mellert G, Zellmeier E, Schneider S, Kakadia PM, et al. Rapid and sensitive screening for CEBPA mutations in acute myeloid leukaemia. Br J Haematol. 2008 Oct;143(2):230–9.

9. Chi Y, Lindgren V, Quigley S, Gaitonde S. Acute myelogenous leukemia with t(6;9)(p23;q34) and marrow basophilia: an overview. Arch Pathol Lab Med. 2008 Nov;132(11):1835–7.
